# 2-Phenyl­acetic acid–(*E*,*E*)-4,4’-(hydra­zinediylidene)dipyridine (2/1)

**DOI:** 10.1107/S1600536810037694

**Published:** 2010-09-25

**Authors:** Hadi D. Arman, Trupta Kaulgud, Edward R. T. Tiekink

**Affiliations:** a Department of Chemistry, The University of Texas at San Antonio, One UTSA Circle, San Antonio, Texas 78249-0698, USA; b Department of Chemistry, University of Malaya, 50603 Kuala Lumpur, Malaysia

## Abstract

The asymmetric unit of the title co-crystal, C_12_H_10_N_4_·2C_8_H_8_O_2_, comprises a single mol­ecule of 2-phenyl­acetic acid and half a mol­ecule of 4-pyridine­aldazine as this is situated about a centre of inversion. Mol­ecules are connected into a three component aggregate *via* O—H⋯N hydrogen bonds. As the carb­oxy­lic acid group is almost normal to the plane through the benzene ring to which it is attached [C—C—C—C = 93.7 (3) °], and the 4-pyridine­aldazine mol­ecule is planar (r.m.s. deviation of the 16 non-H atoms = 0.010 Å), the overall shape of the aggregate is that of an extended chair. In the crystal packing, layers of three component aggregates stack along the *c* axis.

## Related literature

For related studies on co-crystal formation involving the isomeric *n*-pyridine­aldazines, see: Broker *et al.* (2008 ▶); Arman *et al.* (2010 ▶).
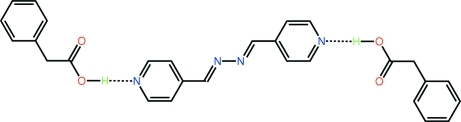


## Experimental

### 

#### Crystal data

C_12_H_10_N_4_·2C_8_H_8_O_2_
*M* *_r_* = 482.53
Monoclinic, 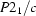

*a* = 11.677 (7) Å
*b* = 4.425 (2) Å
*c* = 23.587 (13) Å
β = 95.475 (8)°
*V* = 1213.2 (11) Å^3^
*Z* = 2
Mo *K*α radiation
μ = 0.09 mm^−1^
*T* = 98 K
0.40 × 0.16 × 0.05 mm

#### Data collection

Rigaku AFC12/SATURN724 diffractometer
5399 measured reflections
2117 independent reflections
1735 reflections with *I* > 2σ(*I*)
*R*_int_ = 0.056

#### Refinement

*R*[*F* ^2^ > 2σ(*F* ^2^)] = 0.068
*wR*(*F* ^2^) = 0.152
*S* = 1.14
2117 reflections
166 parameters
H atoms treated by a mixture of independent and constrained refinement
Δρ_max_ = 0.18 e Å^−3^
Δρ_min_ = −0.21 e Å^−3^

### 

Data collection: *CrystalClear* (Molecular Structure Corporation & Rigaku, 2005 ▶); cell refinement: *CrystalClear*; data reduction: *CrystalClear*; program(s) used to solve structure: *SHELXS97* (Sheldrick, 2008 ▶); program(s) used to refine structure: *SHELXL97* (Sheldrick, 2008 ▶); molecular graphics: *DIAMOND* (Brandenburg, 2006 ▶); software used to prepare material for publication: *publCIF* (Westrip, 2010 ▶).

## Supplementary Material

Crystal structure: contains datablocks global, I. DOI: 10.1107/S1600536810037694/om2363sup1.cif

Structure factors: contains datablocks I. DOI: 10.1107/S1600536810037694/om2363Isup2.hkl

Additional supplementary materials:  crystallographic information; 3D view; checkCIF report

## Figures and Tables

**Table 1:** Hydrogen-bond geometry (Å, °)

*D*—H⋯*A*	*D*—H	H⋯*A*	*D*⋯*A*	*D*—H⋯*A*
O1—H1*o*⋯N1	0.96 (4)	1.70 (4)	2.653 (3)	175 (3)

**Fig. 1.**
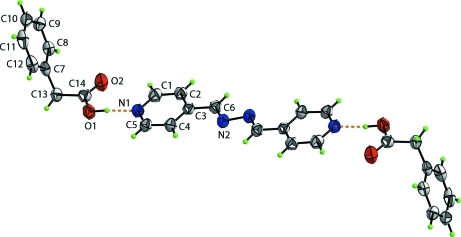
The three component aggregate in the 2:1 co-crystal formed between 2-phenylacetic acid and 4-pyridinealdazine showing atom-labelling scheme and displacement ellipsoids at the 70% probability level. Unlabelled atoms are related by the symmetry operation 1 - x, 1 - y, -z. The view highlights the extended chair conformation and the O—H···N hydrogen bonds (shown as orange dashed lines).

**Fig. 2.**
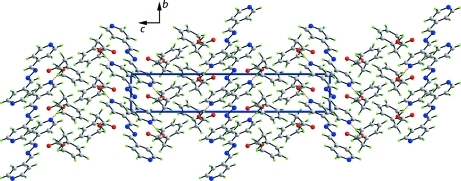
A view in projection down the a axis showing the stacking of layers of three component aggregates along c. The O—H···N hydrogen bonds are shown as orange dashed lines.

## supplementary crystallographic information

### Comment

As a continuation of studies into the phenomenon of co-crystallization of the isomeric n-pyridinealdazines (Broker *et al.*, 2008; Arman *et al.*, 2010), the co-crystallization of 2-phenylacetic acid and 4-pyridinealdazine was investigated. This lead to the isolation of the title 2/1 co-crystal.

The asymmetric unit comprises a molecule of 2-phenylacetic acid and half a molecule of 4-pyridinealdazine, with the latter disposed about a centre of inversion. The constituents are connected by O—H···N hydrogen bonds, Table 1, to generate a centrosymmetric three component aggregate, Fig. 1. The 2-phenylacetic acid molecule is non-planar as seen in the value of the C12–C7–C13—C14 torsion angle of 93.7 (3) °. By contrast, the 4-pyridinealdazine molecule is planar with the r.m.s. deviation of the 16 non-hydrogen atoms being 0.010 Å. Hence, the three component aggregate has the shape of an extended chair, Fig. 1.

In the crystal packing, the three component aggregates pack into layers that stack along the *c* axis, Fig. 2. There are no specific additional intermolecular interactions of note.

### Experimental

Yellow crystals of (I) were isolated from the 2/1 co-crystallization of 2-phenylacetic acid (Sigma Aldrich) and 4-[(1*E*)-[(*E*)-2-(pyridin-4-ylmethylidene)hydrazin-1- ylidene]methyl]pyridine (Sigma Aldrich) in ethanol, m.p. 395–397 K. IR assignment (cm^-1^): 2923 (ν C—H); 2428 (ν O—H); 1693 (ν C═O); 1602 (ν C═N); 1492,1453, 1409 (ν C–C (aromatic)); 1306 (ν C—N); 817, 716 (δ C—H).

### Refinement

C-bound H-atoms were placed in calculated positions (C–H 0.95–0.99 Å) and were included in the refinement in the riding model approximation with *U*_iso_(H) set to 1.2*U*_eq_(C). The O-bound H-atom was located in a difference Fourier map and was refined with *U*_iso_(H) = 1.5*U*_eq_(O).

### Crystal data

**Table:**

C_12_H_10_N_4_·2C_8_H_8_O_2_	*F*(000) = 508
*M**_r_* = 482.53	*D*_x_ = 1.321 Mg m^−^^3^
Monoclinic, *P*2_1_/*c*	Mo *K*α radiation, λ = 0.71073 Å
Hall symbol: -P 2ybc	Cell parameters from 4281 reflections
*a* = 11.677 (7) Å	θ = 2.4–40.2°
*b* = 4.425 (2) Å	µ = 0.09 mm^−^^1^
*c* = 23.587 (13) Å	*T* = 98 K
β = 95.475 (8)°	Plate, yellow
*V* = 1213.2 (11) Å^3^	0.40 × 0.16 × 0.05 mm
*Z* = 2	

### Data collection

**Table:**

Rigaku AFC12K/SATURN724 diffractometer	1735 reflections with *I* > 2σ(*I*)
Radiation source: fine-focus sealed tube	*R*_int_ = 0.056
graphite	θ_max_ = 25.0°, θ_min_ = 2.4°
ω scans	*h* = −13→13
5399 measured reflections	*k* = −5→4
2117 independent reflections	*l* = −28→28

### Refinement

**Table:**

Refinement on *F*^2^	Primary atom site location: structure-invariant direct methods
Least-squares matrix: full	Secondary atom site location: difference Fourier map
*R*[*F*^2^ > 2σ(*F*^2^)] = 0.068	Hydrogen site location: inferred from neighbouring sites
*wR*(*F*^2^) = 0.152	H atoms treated by a mixture of independent and constrained refinement
*S* = 1.14	*w* = 1/[σ^2^(*F*_o_^2^) + (0.0468*P*)^2^ + 0.7865*P*] where *P* = (*F*_o_^2^ + 2*F*_c_^2^)/3
2117 reflections	(Δ/σ)_max_ = 0.001
166 parameters	Δρ_max_ = 0.18 e Å^−^^3^
0 restraints	Δρ_min_ = −0.21 e Å^−^^3^

### Special details

**Table:**

Geometry. All s.u.'s (except the s.u. in the dihedral angle between two l.s. planes) are estimated using the full covariance matrix. The cell s.u.'s are taken into account individually in the estimation of s.u.'s in distances, angles and torsion angles; correlations between s.u.'s in cell parameters are only used when they are defined by crystal symmetry. An approximate (isotropic) treatment of cell s.u.'s is used for estimating s.u.'s involving l.s. planes.
Refinement. Refinement of *F*^2^ against ALL reflections. The weighted *R*-factor *wR* and goodness of fit *S* are based on *F*^2^, conventional *R*-factors *R* are based on *F*, with *F* set to zero for negative *F*^2^. The threshold expression of *F*^2^ > 2σ(*F*^2^) is used only for calculating *R*-factors(gt) *etc*. and is not relevant to the choice of reflections for refinement. *R*-factors based on *F*^2^ are statistically about twice as large as those based on *F*, and *R*- factors based on ALL data will be even larger.

### Fractional atomic coordinates and isotropic or equivalent isotropic displacement parameters (Å^2^)

**Table:**

	*x*	*y*	*z*	*U*_iso_*/*U*_eq_	
N1	0.18146 (19)	1.2809 (5)	0.09616 (10)	0.0316 (6)	
N2	0.4766 (2)	0.6025 (5)	0.01894 (9)	0.0310 (5)	
C1	0.1394 (3)	1.1780 (6)	0.04489 (13)	0.0361 (7)	
H1	0.0651	1.2424	0.0298	0.043*	
C2	0.2001 (2)	0.9811 (6)	0.01301 (12)	0.0321 (6)	
H2	0.1668	0.9083	−0.0227	0.038*	
C3	0.3098 (2)	0.8921 (6)	0.03387 (11)	0.0269 (6)	
C4	0.3540 (2)	1.0017 (6)	0.08676 (12)	0.0303 (6)	
H4	0.4290	0.9457	0.1024	0.036*	
C5	0.2874 (2)	1.1929 (6)	0.11616 (12)	0.0305 (6)	
H5	0.3183	1.2657	0.1523	0.037*	
C6	0.3752 (2)	0.6842 (6)	0.00068 (11)	0.0291 (6)	
H6	0.3414	0.6098	−0.0348	0.035*	
O1	0.05896 (17)	1.6320 (5)	0.15872 (9)	0.0411 (6)	
H1o	0.099 (3)	1.503 (8)	0.1346 (15)	0.062*	
O2	−0.07727 (18)	1.5893 (5)	0.08618 (9)	0.0463 (6)	
C7	−0.2197 (2)	1.6934 (6)	0.18430 (11)	0.0291 (6)	
C8	−0.3183 (2)	1.6647 (6)	0.14658 (11)	0.0301 (6)	
H8	−0.3235	1.7709	0.1114	0.036*	
C9	−0.4091 (2)	1.4834 (6)	0.15960 (12)	0.0345 (7)	
H9	−0.4755	1.4651	0.1333	0.041*	
C10	−0.4032 (3)	1.3282 (6)	0.21105 (13)	0.0393 (7)	
H10	−0.4653	1.2043	0.2201	0.047*	
C11	−0.3063 (3)	1.3562 (6)	0.24861 (13)	0.0417 (8)	
H11	−0.3017	1.2502	0.2838	0.050*	
C12	−0.2149 (3)	1.5378 (6)	0.23580 (12)	0.0368 (7)	
H12	−0.1488	1.5557	0.2624	0.044*	
C13	−0.1189 (2)	1.8779 (6)	0.16826 (13)	0.0358 (7)	
H13A	−0.0727	1.9483	0.2032	0.043*	
H13B	−0.1471	2.0576	0.1462	0.043*	
C14	−0.0443 (2)	1.6869 (6)	0.13282 (12)	0.0325 (6)	

### Atomic displacement parameters (Å^2^)

**Table:**

	*U*^11^	*U*^22^	*U*^33^	*U*^12^	*U*^13^	*U*^23^
N1	0.0300 (13)	0.0327 (12)	0.0328 (13)	0.0018 (10)	0.0066 (11)	−0.0014 (10)
N2	0.0320 (13)	0.0324 (12)	0.0296 (13)	0.0028 (10)	0.0081 (10)	−0.0008 (10)
C1	0.0324 (16)	0.0392 (15)	0.0366 (17)	0.0032 (13)	0.0027 (13)	−0.0021 (13)
C2	0.0295 (15)	0.0381 (15)	0.0284 (15)	0.0013 (12)	0.0017 (12)	−0.0028 (12)
C3	0.0288 (14)	0.0262 (13)	0.0265 (14)	−0.0012 (11)	0.0071 (12)	0.0009 (11)
C4	0.0300 (15)	0.0309 (14)	0.0303 (15)	0.0026 (11)	0.0037 (12)	0.0007 (12)
C5	0.0323 (16)	0.0342 (14)	0.0258 (14)	0.0001 (12)	0.0061 (12)	0.0016 (12)
C6	0.0334 (16)	0.0290 (13)	0.0252 (14)	−0.0010 (12)	0.0046 (12)	0.0013 (11)
O1	0.0295 (11)	0.0523 (13)	0.0409 (12)	0.0074 (9)	0.0006 (9)	−0.0177 (10)
O2	0.0408 (13)	0.0626 (14)	0.0345 (12)	0.0149 (11)	−0.0011 (10)	−0.0111 (11)
C7	0.0319 (15)	0.0282 (13)	0.0279 (14)	0.0070 (11)	0.0066 (12)	−0.0058 (11)
C8	0.0347 (16)	0.0308 (14)	0.0251 (14)	0.0036 (12)	0.0038 (12)	−0.0017 (11)
C9	0.0326 (16)	0.0350 (14)	0.0363 (17)	0.0025 (12)	0.0054 (13)	−0.0028 (13)
C10	0.0455 (19)	0.0337 (15)	0.0418 (18)	0.0001 (13)	0.0197 (15)	−0.0055 (13)
C11	0.062 (2)	0.0369 (16)	0.0276 (16)	0.0122 (15)	0.0146 (15)	0.0034 (13)
C12	0.0443 (18)	0.0388 (15)	0.0265 (15)	0.0119 (13)	−0.0004 (13)	−0.0066 (12)
C13	0.0316 (16)	0.0351 (15)	0.0413 (18)	0.0018 (12)	0.0071 (13)	−0.0111 (13)
C14	0.0330 (16)	0.0330 (14)	0.0318 (16)	−0.0003 (12)	0.0054 (13)	0.0003 (12)

### Geometric parameters (Å, °)

**Table:**

N1—C5	1.339 (3)	O2—C14	1.210 (3)
N1—C1	1.341 (4)	C7—C8	1.392 (4)
N2—C6	1.273 (3)	C7—C12	1.393 (4)
N2—N2^i^	1.419 (4)	C7—C13	1.510 (4)
C1—C2	1.389 (4)	C8—C9	1.387 (4)
C1—H1	0.9500	C8—H8	0.9500
C2—C3	1.385 (4)	C9—C10	1.390 (4)
C2—H2	0.9500	C9—H9	0.9500
C3—C4	1.391 (4)	C10—C11	1.374 (4)
C3—C6	1.468 (4)	C10—H10	0.9500
C4—C5	1.380 (4)	C11—C12	1.392 (4)
C4—H4	0.9500	C11—H11	0.9500
C5—H5	0.9500	C12—H12	0.9500
C6—H6	0.9500	C13—C14	1.520 (4)
O1—C14	1.321 (3)	C13—H13A	0.9900
O1—H1o	0.96 (4)	C13—H13B	0.9900
			
C5—N1—C1	117.7 (2)	C9—C8—C7	120.9 (3)
C6—N2—N2^i^	111.7 (3)	C9—C8—H8	119.5
N1—C1—C2	122.6 (3)	C7—C8—H8	119.5
N1—C1—H1	118.7	C8—C9—C10	120.3 (3)
C2—C1—H1	118.7	C8—C9—H9	119.9
C3—C2—C1	119.3 (3)	C10—C9—H9	119.9
C3—C2—H2	120.4	C11—C10—C9	119.2 (3)
C1—C2—H2	120.4	C11—C10—H10	120.4
C2—C3—C4	118.1 (2)	C9—C10—H10	120.4
C2—C3—C6	119.9 (2)	C10—C11—C12	120.9 (3)
C4—C3—C6	122.0 (2)	C10—C11—H11	119.6
C5—C4—C3	119.1 (3)	C12—C11—H11	119.6
C5—C4—H4	120.5	C11—C12—C7	120.4 (3)
C3—C4—H4	120.5	C11—C12—H12	119.8
N1—C5—C4	123.2 (3)	C7—C12—H12	119.8
N1—C5—H5	118.4	C7—C13—C14	109.8 (2)
C4—C5—H5	118.4	C7—C13—H13A	109.7
N2—C6—C3	120.8 (2)	C14—C13—H13A	109.7
N2—C6—H6	119.6	C7—C13—H13B	109.7
C3—C6—H6	119.6	C14—C13—H13B	109.7
C14—O1—H1O	108 (2)	H13A—C13—H13B	108.2
C8—C7—C12	118.3 (3)	O2—C14—O1	123.5 (3)
C8—C7—C13	120.4 (3)	O2—C14—C13	123.3 (3)
C12—C7—C13	121.2 (3)	O1—C14—C13	113.2 (2)
			
C5—N1—C1—C2	1.5 (4)	C13—C7—C8—C9	176.6 (2)
N1—C1—C2—C3	−1.8 (4)	C7—C8—C9—C10	0.5 (4)
C1—C2—C3—C4	0.9 (4)	C8—C9—C10—C11	−0.2 (4)
C1—C2—C3—C6	−180.0 (2)	C9—C10—C11—C12	0.2 (4)
C2—C3—C4—C5	0.1 (4)	C10—C11—C12—C7	−0.4 (4)
C6—C3—C4—C5	−179.0 (2)	C8—C7—C12—C11	0.6 (4)
C1—N1—C5—C4	−0.4 (4)	C13—C7—C12—C11	−176.6 (2)
C3—C4—C5—N1	−0.3 (4)	C8—C7—C13—C14	−83.5 (3)
N2^i^—N2—C6—C3	178.9 (2)	C12—C7—C13—C14	93.7 (3)
C2—C3—C6—N2	179.3 (2)	C7—C13—C14—O2	64.5 (4)
C4—C3—C6—N2	−1.7 (4)	C7—C13—C14—O1	−113.9 (3)
C12—C7—C8—C9	−0.7 (4)		

Symmetry codes: (i) −*x*+1, −*y*+1, −*z*.

### Hydrogen-bond geometry (Å, °)

**Table:**

*D*—H···*A*	*D*—H	H···*A*	*D*···*A*	*D*—H···*A*
O1—H1o···N1	0.96 (4)	1.70 (4)	2.653 (3)	175 (3)
